# Femoral Nerve Injury After Resection of a Retroperitoneal Schwannoma Arising From the Femoral Nerve

**DOI:** 10.7759/cureus.104345

**Published:** 2026-02-26

**Authors:** Soichiro Honda, Kaoru Tada, Mika Akahane, Akari Mori, Satoru Demura

**Affiliations:** 1 Department of Orthopaedic Surgery, Graduate School of Medical Sciences, Kanazawa University, Kanazawa, JPN

**Keywords:** an autologous nerve graft, femoral nerve injury, retropritoneal, schwannnoma, sural nerve

## Abstract

This report describes a case of femoral nerve injury resulting from retroperitoneal tumor resection, which was successfully treated with an autologous nerve graft using the sural nerve. The patient, a 27-year-old woman, had gait disturbance and sensory loss in her lower limbs following surgery and was referred to the department at four months postoperatively. Lower extremity muscle strength testing showed that the left quadriceps muscle was decreased to 0-1 on Manual Muscle Testing (MMT), and there was sensory loss in front of the thigh to the medial side of the lower leg. The surgery was performed using an ilioinguinal approach, and the cable graft made from the sural nerve was implanted. At three years postoperatively, the quadriceps muscle had recovered to MMT3, and the patient was able to walk stably without crutches. An autologous nerve graft is a useful procedure for patients with good recovery prospects.

## Introduction

Retroperitoneal tumors are uncommon, and schwannomas represent the most frequent neurogenic subtype [1]. Although generally benign, these tumors are difficult to diagnose preoperatively because they lack specific clinical or radiological features [2]. Magnetic resonance imaging (MRI) is the preferred modality for evaluation, but definitive diagnosis is often achieved only after histopathological confirmation.

Surgical excision is usually indicated for symptomatic lesions, both to obtain a diagnosis and to relieve mass effect [3]. However, since retroperitoneal schwannomas may arise from major nerves, surgery carries a risk of iatrogenic nerve injury, which can cause severe motor and sensory deficits [4]. In particular, femoral nerve injury compromises knee extension by weakening the quadriceps femoris, thereby markedly reducing ambulatory function.

Several reconstructive options have been described for such injuries, including autologous sural nerve grafting and obturator nerve transfer [5-7]. Autologous nerve graft remains a reliable method in young patients with favorable recovery potential. Here, we report a case of femoral nerve injury following resection of a retroperitoneal schwannoma, successfully treated with an autologous sural nerve graft.

## Case presentation

**Figure 1:**
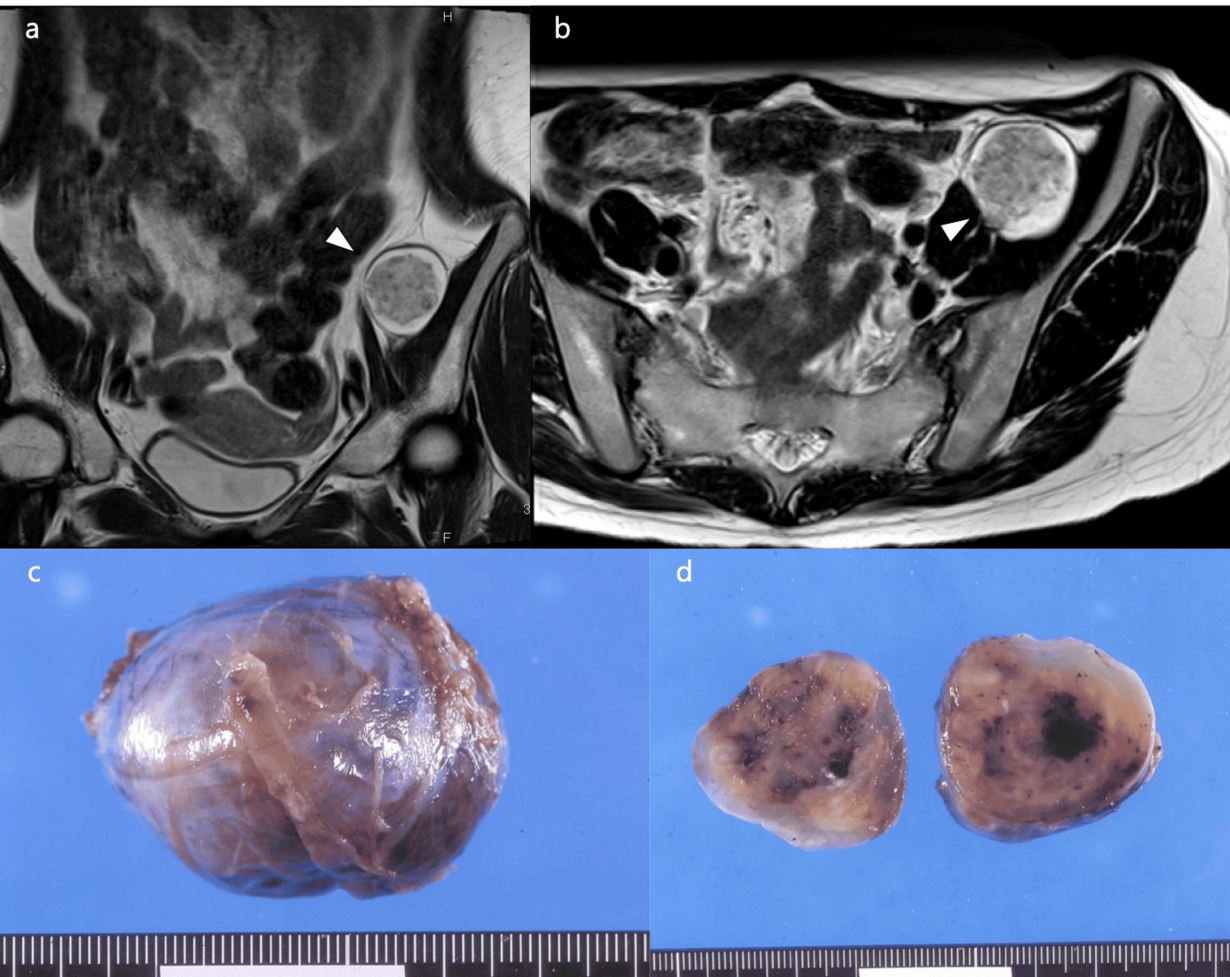
MRI images and the excised tumor (a) T2-weighted coronal image, white arrowheads indicate tumor; (b) T2-weighted axial image, white arrowheads indicate tumor; (c) Excised tumor; (d) Cross-section of excised tumor divided into two sections.

A 27-year-old female patient underwent laparoscopic resection of a left retroperitoneal tumor, which was pathologically confirmed as a schwannoma (Figure 1). Although the previous hospital suspected a neurogenic tumor, vascular tumor, or gastrointestinal stromal tumor preoperatively, intraoperative neuromonitoring was not performed.

Postoperatively, the patient developed gait disturbance and sensory loss in her lower limb. Although gait training rehabilitation was performed, no recovery was observed, and she was referred to our department four months later.

**Figure 2:**
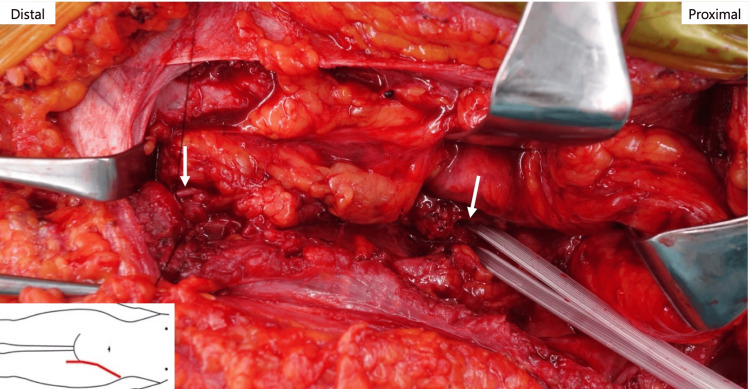
Femoral nerve defect measuring 75 mm in length White arrows indicate distal and proximal nerve stumps

At the initial visit, she was unable to walk stably without crutches due to her left knee buckling. Manual muscle testing (MMT) revealed that the left quadriceps muscle strength was 0-1, and sensory loss was present from the anterior thigh to the medial side of the lower leg. Based on these findings, femoral nerve injury due to tumor resection was diagnosed, and an autologous nerve graft was planned. Through an ilioinguinal approach, the retroperitoneum was dissected, and the femoral nerve was identified at the posterior surface of the iliopsoas muscle (Figure 2).

**Figure 3:**
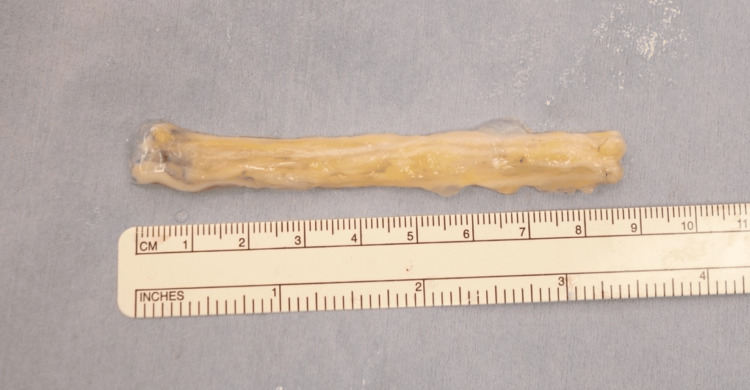
Cable graft Bilateral sural nerves were harvested, and cable graft was made

Bilateral sural nerves were harvested, measuring a total of 300 × 2 mm, and bundled with fibrin glue to create an 85-mm cable graft (Figure 3).

**Figure 4:**
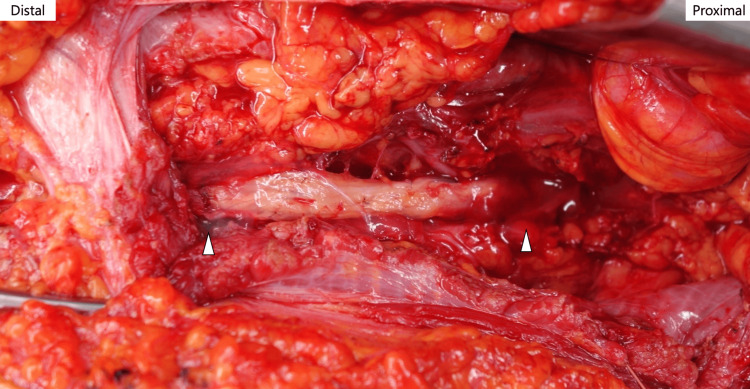
The cable graft was implanted into the nerve defect and fibrin glue was spread around the graft White arrowheads indicates stitched site.

The graft was implanted to bridge the defect, and fibrin glue was applied around the graft to complete the repair (Figure 4).

**Figure 5:**
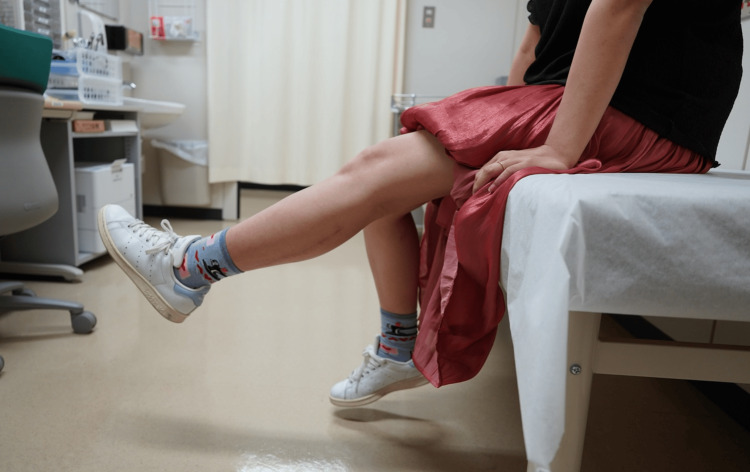
Knee extension three years postoperatively Patient was able to achieve automatic extension of the left knee joint.

For the first two weeks after surgery, the patient practiced walking while carefully avoiding hip hyperextension. She continued outpatient rehabilitation two to three times a month for about one year postoperatively, and thereafter engaged in home-based strengthening exercises under occasional supervision by a physical therapist. At two years postoperatively, she still had difficulty with automatic knee extension, but by three years the quadriceps muscle had recovered to MMT3, allowing voluntary extension of the knee (Figure 5). She was able to walk stably without crutches. Although she experienced numbness at the sural nerve donor site, the residual symptoms did not affect daily activities, and she was satisfied with the outcome.

## Discussion

Several reports have described femoral nerve injury following retroperitoneal tumor resection, with treatment strategies including autologous sural nerve grafting and obturator nerve transfer (Table 1) [4-7].

**Table 1:** Summary of reports of femoral nerve injury following retroperitoneal tumor resection *MMT  of the quadriceps at the last follow-up MMT: manual muscle test

Surgical procedure	Authors	Age (years)	Sex	Waiting period for surgery (months)	Defects of nerve (mm)	Length of graft (mm)	Duration of follow-up (years)	Outcome (MMT*)
Auto nerve graft	Current study	27	F	4	80	85	3.5	4
	Tsuchihara et al., 2008 [4]	40	M	1	120	130	2.5	4
		57	M	3	70	140	3	3
Nerve transfer	Campbell et al., 2010 [5]	45	F	3	150	-	2	4
	Tung et al., 2012 [6]	31	F	1	-	-	2.5	4
		39	F	5	-	-	3	4+
	O'Brien et al., 2021 [7]	27	F	8	-	-	2	3

In previous reports of autologous nerve graft, quadriceps strength recovered to MMT4 or greater, even when nerve defects exceeded 100 mm, particularly in younger patients and when reconstruction was performed early [4,8]. By harvesting the sural nerve, a graft length of up to 140 mm can be achieved [9]. The main drawback of autologous graft is donor site morbidity.

Regarding motor nerve recovery, older age and delayed treatment are associated with poorer outcomes [10]. When the nerve graft length exceeds 10 cm, irreversible remodeling at the neuromuscular junction may occur before reinnervation. Accordingly, we propose that an autologous nerve graft is indicated for younger patients, those treated within six months of injury, and those with nerve defects ≤10 cm.

Tada et al. reported that donor site complaints may persist, but symptoms are generally mild, and patient satisfaction remains high when recovery is achieved [11]. In the present case, although the patient experienced numbness in the sural nerve distribution of both lower legs, she has been able to live without difficulty due to satisfactory muscle strength recovery.

Obturator nerve transfer has been proposed as an alternative, enabling reinnervation with a single suture close to the motor point of the quadriceps [5,6]. Although there is concern about the weakness of the hip adductor muscles, O’Brien et al. described a technique that preserves proximal obturator innervation, reducing this drawback [7]. This approach may therefore be a reasonable alternative in selected cases.

In our patient, an 85-mm autologous sural nerve graft was chosen because of her young age and the feasibility of reconstruction. The favorable outcome supports the indication of autologous nerve grafting for younger patients with nerve defects of approximately 100 mm, while obturator nerve transfer may be more suitable for elderly patients or cases where a graft is not feasible.

## Conclusions

In this case, an 85-mm autologous sural nerve graft was performed to reconstruct a femoral nerve defect following retroperitoneal tumor resection, resulting in a favorable functional recovery. This case supports the usefulness of autologous nerve grafting for young patients with nerve defects of approximately 100 mm. For elderly patients or cases in which autologous grafting is not feasible, obturator nerve transfer may be considered as an alternative.
